# Harnessing mechanobiology for kidney organoid research

**DOI:** 10.3389/fcell.2023.1273923

**Published:** 2023-11-24

**Authors:** Zarina Nauryzgaliyeva, Iphigénie Goux Corredera, Elena Garreta, Nuria Montserrat

**Affiliations:** ^1^ Institute for Bioengineering of Catalonia (IBEC), The Barcelona Institute of Science and Technology (BIST), Barcelona, Spain; ^2^ Pluripotency for Organ Regeneration, Institute for Bioengineering of Catalonia (IBEC), The Barcelona Institute of Science and Technology (BIST), University of Barcelona, Barcelona, Spain; ^3^ Centro de Investigación Biomédica en Red en Bioingeniería, Biomateriales y Nanomedicina, Institució Catalana de Recerca i Estudis Avançats (ICREA), Barcelona, Spain

**Keywords:** mechanobiology, organoids, hPSCs, nephrogenesis, development

## Abstract

Recently, organoids have emerged as revolutionizing tools with the unprecedented potential to recreate organ-specific microanatomy *in vitro*. Upon their derivation from human pluripotent stem cells (hPSCs), organoids reveal the blueprints of human organogenesis, further allowing the faithful recapitulation of their physiology. Nevertheless, along with the evolution of this field, advanced research exposed the organoids’ shortcomings, particularly regarding poor reproducibility rates and overall immatureness. To resolve these challenges, many studies have started to underscore the relevance of mechanical cues as a relevant source to induce and externally control hPSCs differentiation. Indeed, established organoid generation protocols from hPSCs have mainly relyed on the biochemical induction of fundamental signalling pathways present during kidney formation in mammals, whereas mechanical cues have largely been unexplored. This review aims to discuss the pertinence of (bio) physical cues within hPSCs-derived organoid cultures, while deciphering their effect on morphogenesis. Moreover, we will explore state-of-the-art mechanobiology techniques as revolutionizing means for understanding the underlying role of mechanical forces in biological processes in organoid model systems.

## Introduction

The kidney plays a vital role in regulating bodily fluids and maintaining their composition. It performs crucial tasks such as blood filtration, hormone regulation, and waste removal, all of which are essential for maintaining organism homeostasis. The kidney’s remarkable anatomy enables it to carry out these functions effectively: its highly vascularized structure allows for efficient filtration from the blood vessels, while its connection to the urinary tract facilitates the excretion of waste in the form of urine. These processes are carefully regulated by signalling hormones within the body, which are essential for maintaining blood pressure, electrolyte concentration and acid-base balance.

The kidneys contain approximately 1 million nephrons, which are the functional units (Bertram et al., 2011). Each nephron consists of two major components: the renal corpuscle, responsible for blood filtration, and tubular structures that facilitate the uptake and secretion of solutes. At the distal end of each nephron, waste and excess water are drained into a network of collecting ducts, which eventually converge into the ureter.

In humans, the formation of nephrons (nephrogenesis), is completed around 36 weeks of pregnancy, ensuring the kidney’s complexity is established by birth. Throughout this developmental time, various environmental cues and genetic factors guide the precise localization and formation of tissue components within the organ. While the genetic and biochemical cues involved in kidney development are well understood, the role of mechanical cues has remained largely unexplored.

Given the kidney’s intrinsic complexity and its vital role in maintaining overall organism homeostasis, it has gathered significant interest in the scientific community. Researchers have been particularly focused on unravelling the morphogenetic principles that govern kidney development from early embryonic stages until birth. Advancements in developmental studies, including the use of human pluripotent stem cells- (hPSCs) or adult stem cell-derived organoids, have pushed the boundaries of our understanding of organogenesis and disease progression in this complex organ. However, despite these major breakthroughs, the precise mechanisms underlying the (bio) mechanical machinery that contributes to renal fate specification and the overall tissue organization *in vivo* remain unknown.

As the recognition of mechanical cues in development grows, there is an increasing demand for sophisticated bioengineering solutions to understand the mechanical signals and responses involved in organogenesis. Mechanobiological techniques, as reviewed in (Gómez-González et al., 2020), can help uncover the role of mechanical cues in tissue morphogenesis. At the same time, the generation of a more representative developmental architecture using hPSCs-derived organoids, as discussed in (Garreta et al., 2021), holds tremendous promise for gaining a deeper understanding of the interplay between the genetic and (bio) mechanical machinery contributing to organogenesis.

This review aims to summarize current knowledge on mammalian kidney development and provide insights into the state-of-the-art methodologies for *in vitro* kidney organoid differentiation, which pose as valuable tools for studying development and disease. The review will also discuss what is known so far regarding the presence of mechanical cues during embryo and organ development and will highlight state-of-the-art techniques commonly employed in the field of tissue mechanics to probe cell-to-tissues responses. At the same time, here we aim to provide a comprehensive view on how these tools may be translated to understand kidney morphogenesis exploiting hPSCs-derived kidney organoid technology. Finally, the review will explore the rising need for more interdisciplinarity between the fields of classical developmental biology and mechanobiology, which can significantly enhance our understanding of organ development and disease progression in humans.

## The permanent adult kidney rises from the metanephros

The development of the adult mammalian kidney begins during embryogenesis with the formation of three precursor structures: pronephros, mesonephros and metanephros. The prior two are transient, yet still necessary structures, the absence of which have been associated with renal agenesis. The metanephros on the other hand is the permanent structure that gives rise to the functional adult kidney (Reidy and Rosenblum, 2009). Primitive urinary system commitment occurs at the blastula-stage of embryonic development, where the blastocyst undergoes an invagination, referred to as the primitive streak (PS). At those early stages of development, antero-posterior (A-P) patterning information is conveyed by the movement of cells through the PS at different time points that consequently affects the expression of kidney genes. Importantly, other kidney-inductive signals are present along the whole axis, including anterior non-kidney-generating regions. Then, the posterior end of the PS, as largely demonstrated by making use of embryonic mouse experiments [reviewed in (Dressler, 2009; Halt and Vainio, 2014)] further evolves to the Intermediate Mesoderm (IM), from where the urogenital system -the kidneys, the gonads, and their respective duct systems-will be derived. Upon polarization, the IM will be segmented into the anterior IM (AIM) and posterior IM (PIM). The AIM eventually epithelializes into the Wolffian duct, also known as the mesonephric duct which are tubular structures leading to the cloaca (future bladder). In humans, at the 5th week of gestation, a small protrusion of the mesonephric duct is observed, named the ureteric bud (UB), indicating the initiation of the metanephros formation. This structure is rapidly enclosed by cells from the metanephric mesenchyme (MM) lineage which further aggregate surrounding the bud’s ends, forming cap-like structures, namely, the cap mesenchyme. From this stage onwards, reciprocal interactions between cells from the UB and MM lineage are established, guiding their co-development. MM cells produce GDNF, ultimately stimulating UB branching morphogenesis to form a urine draining tubular network. In parallel, canonical Wnt signals from UB cells to the MM population induces the mesenchymal-to-epithelial transition (MET) of nephron progenitor cells (NPCs). Upon activation of the Wnt pathway, differentiated NPCs further cluster together forming lumen-presenting spherical aggregates termed renal vesicles (RVs). These are then polarized, elongated and undergo a series of structural changes to eventually segment into the different sections of the mature nephron (Dressler, 2006; Lindström and Tran, 2018b; Khoshdel Rad et al., 2020).

These RV structures elongate into polarized comma-shaped bodies, then S-shaped bodies, readily connected to the UB-derived collecting duct. S-shaped bodies reportedly evolve into mature nephrons, exhibiting glomerulus and tubular structures, including the proximal tubule, loop of Henle and distal tubule. All these structural changes are known to be guided by the genetic blueprint. In humans nephrogenesis repeats until shortly before birth.

Like any other developmental process, kidney organogenesis exhibits a remarkable sensitivity. Indeed, disruptions or impairment at any stage of kidney morphogenesis can precipitate a cascade of severe inborn conditions, ultimately known as congenital anomalies of the kidneys and urinary tract (CAKUT). As such, CAKUT encompasses a family of urinary system malformation at birth, including kidney defects such as renal agenesis or hypoplasia, as well as ureteric malfunctions (Nicolaou et al., 2015). These anomalies, although generally associated with mutations in specific genes involved in the urinary system formation, may also be due to improper physical interactions between UB and MM cells (Jain and Chen, 2019). For instance, supernumerary kidneys, which present multiple ureters, arise upon impaired UB and MM interaction, where UB bifurcation occurs prior MM invasion. In the case of kidney hypoplasia, which manifests as small kidneys with low nephron number, reduced branching morphogenesis events of the UB have been recorded.

Several CAKUT manifestations have been reported to be clinically associated with hypertension, ultimately leading to, in certain occasions, end stage renal disease (ESRD). There is therefore an urge to understand the mechanisms underlying CAKUT anomalies in the human context. Indeed, several works have been developed in knock-out animal models, however, the phenotypical manifestation of the pathological mutations may vary from species to species (Khan et al., 2022).

## Mammalian kidney development is evolutionarily conserved among species

Mammalian kidney development is a fascinating process that exhibits remarkable evolutionary conservation across species. The study of human-specific kidney development throughout gestation has been challenged by limited access to human embryonic kidney samples and the ethical concerns surrounding their origin. However, this limitation has been overcome by utilizing animal models of lower complexity, such as rats and mice, to investigate the intricate cellular and molecular processes involved in kidney organogenesis. By leveraging the remarkable conservation of embryogenesis across mammalian species, findings from these animal models have provided valuable insights into human kidney development (Cullen-McEwen et al., 2015). In fact, these studies have contributed to our understanding of the different stages of kidney morphogenesis and have identified fundamental signalling pathways, including the canonical Wnt pathway, that play a crucial role in this process (Carroll et al., 2005; Lindström and Tran, 2018b).

Comparison of embryonic kidney development across diverse mammalian species has revealed high levels of similarity. Notably, studies have documented shared morphological features between mouse and human embryonic and adult tissues, demonstrating clear parallels in tissue architecture at species-specific gestational timepoints (Lindström and Tran, 2018b). The blueprint of nephron morphology and positioning relative to other cell lineages within the kidney is conserved between both species. Nephron-patterning events also exhibit strong conservation, as observed through immunocytochemistry studies: the polarization of renal vesicles, primitive nephron-oriented structures, segmentation into proximal and distal sections can all be observed in mammalian species.

Despite the diversity of mammalian species, the fundamental principles and mechanisms governing kidney development remain remarkably similar. Indeed, our current understanding of kidney organogenesis has been derived from mouse and rat embryonic kidney samples. Shared anatomy, patterning and signalling pathways have emphasized the utility of mouse embryonic models in recapitulating kidney development when human samples are unavailable. And while the overall process of embryonic kidney development remains evolutionarily conserved among mammalian species, inter-species molecular and anatomical variations still exist (O’Brien et al., 2016; Lindström and Tran, 2018b). Indeed, some divergent features have been observed when comparing mouse to human embryonic nephrogenesis (O’Brien et al., 2016). For example, only a minority of previously established mouse anchor genes, which are expected to distinctly encode kidney structures, display a conserved expression pattern in humans (Lindström and Guo, 2018a). Discrepancies in nephron number have been reported, with mouse kidneys containing approximately 12–16,000 nephrons, while human kidneys include an average of 1 million nephrons. This difference in nephron quantity is due to variations in time of gestation (9 months in humans, approximately 20 days in mice) and the overall kinetics of nephrogenesis (O’Brien and McMahon, 2014).

With regards to inborn conditions affecting the kidney, the mechanisms underlying CAKUT, for instance, have been more challenging to address in non-human models. Importantly, heterozygous or homozygous mutations in mice models have caused either embryonic or postnatal lethality, making it difficult to study the underlying pathophysiology (Stone et al., 2016).

## Human pluripotent stem cells derived organoids: how to model kidney development and disease

The establishment of human embryonic and induced pluripotent stem cell lines (hESCs and hiPSCs, referred as hPSCs) has significantly impacted how scientists study human development and diseases outside of the human body (Thomson et al., 1998; Takahashi and Yamanaka, 2006; Takahashi et al., 2007). The capability of hPSCs to expand indefinitely while preserving pluripotent differentiation features (that is to give rise to cells of the three-germ layer of the embryo *in vivo* and *in vitro*) allows for the interrogation of early stages of cell lineage specification and differentiation. Thanks to this, specific cell culture conditions sustaining and promoting cell differentiation have been identified and are now consistently used as a tool in developmental research. For more than 2 decades researchers world-wide have started to define external conditions aiming to guide hPSCs differentiation while exploiting inherent characteristics of these cells, that are capable of self-organization and symmetry breaking (Armstrong, 1989). While initial studies of hPSCs differentiation promoted early stages of cell lineage specification and commitment in two dimensional culture conditions (Laflamme et al., 2007), seminal studies started to address these questions making use of embryoid bodies (EBs) as surrogates of differentiation and cell specification along the three germ lineages of the human embryo (Yang et al., 2008; Chambers et al., 2009).

A technical evolution of EB culture systems led to the overall explosion of the organoid field from hPSCs. This was based on the seminal work from Yoshiki Sasai on the establishment of the first self-patterned stratified cortical tissues generated by plating EBs in serum-free medium on a coated surface (Eiraku et al., 2008). At the present time, the term “organoid” refers to a three-dimensional (3D) collection of cells that resembles an organ [reviewed in detail in (Lancaster and Knoblich, 2014)]. To achieve resemblance, this 3D structure comprises of several different cell types that are characteristically present in the native organ. *In vivo*, different cell types arise from stem cells through a process of lineage commitment and cell sorting, that allow a spatial organization like an organ during development. *In vitro*, self-organized collection of hPSCs-derived cell types can recapitulate, until certain extent, specific functions of an organ.

**FIGURE 1:**
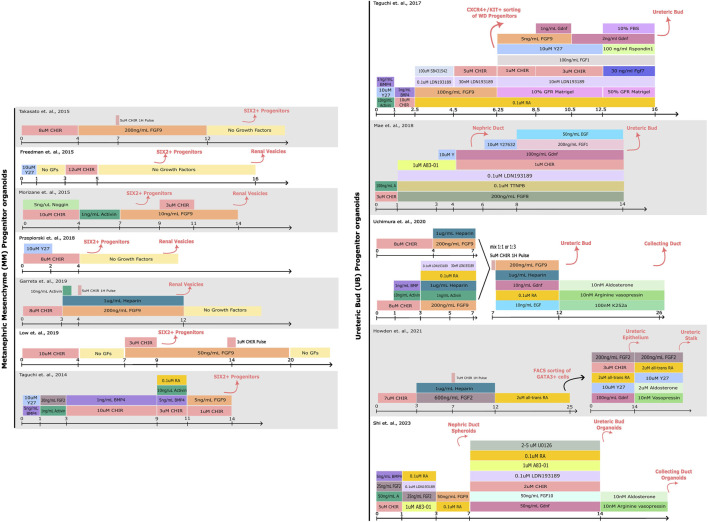
Comparison of protocols for hPSC-derived kidney organoid differentiation. A schematic detailing the compounds used for driving PSCs towards Metanephric Mesenchyme (MM) or Ureteric Bud (UB) progenitor states. The cell types induced by each step of differentiation are denoted above in pink. More specific details regarding the protocols can be found in the corresponding publications (Taguchi et al., 2014; Freedman et al., 2015; Morizane et al., 2015; Takasato et al., 2015; Taguchi and Nishinakamura, 2017; Mae et al., 2018; Przepiorski et al., 2018; Garreta et al., 2019; Low et al., 2019; Uchimura et al., 2020; Howden et al., 2021; Shi et al., 2023).

Cutting-edge research from laboratories around the world have led to the generation of kidney organoids from hPSCs by mimicking *in vitro* renal inductive signals that occur during kidney organogenesis (Xia et al., 2014; Freedman et al., 2015; Morizane et al., 2015; Takasato and ErPei, 2015; Taguchi and Nishinakamura, 2017; Garreta et al., 2019). These studies have revealed specific morphogens and cytokines that can drive the differentiation of PSCs towards a renal specification *ex vivo* (Takasato et al., 2014; 2016; Xia et al., 2014; Morizane et al., 2015; Takasato and ErPei, 2015; Taguchi and Nishinakamura, 2017; Uchimura et al., 2020). Collectively, procedures allowing the generation of organoids resembling separate components of the mammalian kidney have been established (Figure 1), including: nephron progenitor (NP), ureteric bud (UB) and even stromal progenitor (SP) organoids (Zeng et al., 2021; Palakkan et al., 2022; Tanigawa et al., 2022; Vanslambrouck et al., 2022).

Of note, the metanephric adult kidney as an organ comprises a highly complex tissue architecture. The current state-of-the-art of procedures in kidney organoid differentiation show a remarkable resemblance to the first/second trimester human embryonic kidney (Garreta et al., 2019). Yet, these methodologies still lack a higher order of anatomical resemblance, where the developing nephrons would connect with the collecting duct to allow for filtration and re-absorption of nutrients. To address these limitations, specialized tissue culture techniques are being widely explored to drive organoid maturation forward, particularly in terms of nephron development (bioreactors), collective duct development (patterned substrates), and vascularization (microfluidics) that better mimic physiologically-relevant cues (Czerniecki et al., 2018; Przepiorski et al., 2018; Homan et al., 2019; Glass et al., 2020; Shi et al., 2023).

One of the advantages of using hPSC-based organoid systems is that they are highly adaptable for genetic manipulation, making it possible to mimic genetic kidney diseases by introducing alterations in the corresponding genes. Patient-derived and/or gene-edited hPSCs have been an excellent source for studying adult kidney disorders such as chronic kidney disease (CKD) and polycystic kidney diseases (PKD), among others [reviewed in more detail in (Karp et al., 2022; Liu et al., 2022)]. While studies with hPSC- derived organoids capturing the PKD genetic background have been seminal in modelling cyst formation (Freedman et al., 2015) and response to drugs (Cruz et al., 2017; Tran et al., 2022), the immature nature of *in vitro* kidney organoids makes it difficult to model diseases exhibiting adult anomalies. In contrast to chronic-related manifestations, CAKUT maybe effectively studied exploiting hPSCs-derived kidney organoids.

In this regard, CAKUT is an umbrella term encompassing an extensive spectrum of embryonic kidney and urinary tract malformations that affect approximately 1 in 100–500 new-borns (Westland et al., 2021). The phenotypical profile of CAKUT anomalies is quite heterogeneous: the most typical observed phenotypes include hypoplasia, dysplasia, agenesis, ureteral abnormalities among others. More than twenty genes have been identified as liable for the onset of syndromic and non-syndromic CAKUT, potentially explaining the heterogeneous clinical presentation of these congenital anomalies (Nicolaou et al., 2015). Mutations in *PAX2, HNF1B, BMP7, RET, GATA3, SALL1, SIX5, and EYA1* among others developmental genes have been previously reported.

Paired box gene 2 (*PAX2*) is an important developmental gene in kidney organogenesis, expressed throughout nephron differentiation: starting from progenitors to epithelial renal vesicles to distal fragments of the renal tubules. Particularly, in mice, *Pax2* was shown to play an indispensable role in MET of NPs, and the positioning and outgrowth of the ureteric bud (Torres et al., 1995; Brophy et al., 2001). The latter phenomenon is likely responsible for the renal agenesis phenotype observed in mice with an absence of this gene. In humans, on the other hand, frameshift mutations have been associated with the development of renal coloboma (Sanyanusin et al., 1995; Bower et al., 2012; Chang et al., 2022). Interestingly, *in vitro* research performed by Kaku & Nishinakamura et al. has demonstrated that *PAX2* is in fact dispensable for MET in NPs developed from hPSCs (Kaku et al., 2017). In their study, *PAX2-*null hPSCs could still develop into nephron progenitors, and even epithelialize into tubular and glomerular structures. However, while human *PAX2* may not be necessary for MET to occur, it is needed for the proper differentiation of parietal epithelial cells of the glomeruli (Kaku et al., 2017).

Hepatocyte nuclear factor 1—β (*HNF1B*) has been shown to play an important role in nephrogenesis. Studies in zebrafish have identified the role of this transcription factor in nephron patterning and segmentation, where embryos lacking *hnf1ba/b* would not express markers characteristic for distal and proximal segments, yet still form an epithelial tubule (Naylor et al., 2013). Similar findings have been observed in murine and *xenopus* nephrogenesis, where a disruption or overexpression of this gene leads to a failure in SSB patterning and segmentation (Naylor et al., 2013). In humans, mutations in *HNF1B,* together with mutations in *PAX2* account for approximately 15% of known CAKUT cases (Nicolaou et al., 2015). Human iPSC-derived kidney organoids have been used as a platform to mimic congenital kidney anomalies involving *HNF1B* by generating biallelic deletions in the gene. Przepiorski et al. have demonstrated that organoids with disrupted *HNF1B* fail to develop regions positive for markers of proximal and distal tubules, similar to phenotypes previously observed in *Hnf1b* conditionally deficient mice (Przepiorski et al., 2018).

Mutations in the receptor tyrosine kinase *RET* gene are also more commonly found in the spectrum of congenital urinary anomalies like renal agenesis and aplasia. A cohort study found that mutations along the GDNF-RET signalling pathway are present in about 5% of patients with CAKUT and that the nature of the mutations affects the penetrance of CAKUT clinical presentation (Chatterjee et al., 2012). Studies on mutant mice carrying genetic alterations along the *GDNF-Gfra1-Ret* axis have shed light on the expression patterns of *Ret* throughout the kidney and lower urinary tract [reviewed in detail in (Jain, 2009)]. Briefly, *Ret* plays a crucial role in early UB induction and branching, among other roles, without which extreme phenotypes such as renal agenesis or prenatal fatality can be observed. Human PSC-derived UB organoids have been successfully used to mimic congenital anomalies resulting from mutations in *RET*, proving the feasibility of modelling such diseases by *in vitro* genetic editing and 3D culture (Zeng et al., 2021). In their study, the authors have demonstrated how *RET-*null organoids fail to undergo branching and exhibit a phenotype like renal agenesis.

Similarly, hPSC-derived kidney organoid systems have been successfully utilized to model certain podocytopathies by either genetically introducing mutations into wild type PSCs, or by inducing them from patient-specific cells (Kim et al., 2017; Hale et al., 2018). Together, all these studies provide a promising outlook on the use of hPSC-derived kidney organoids as a platform to study early stages of kidney development and diseases, where cases of CAKUT could be of particular interest.

**FIGURE 2:**
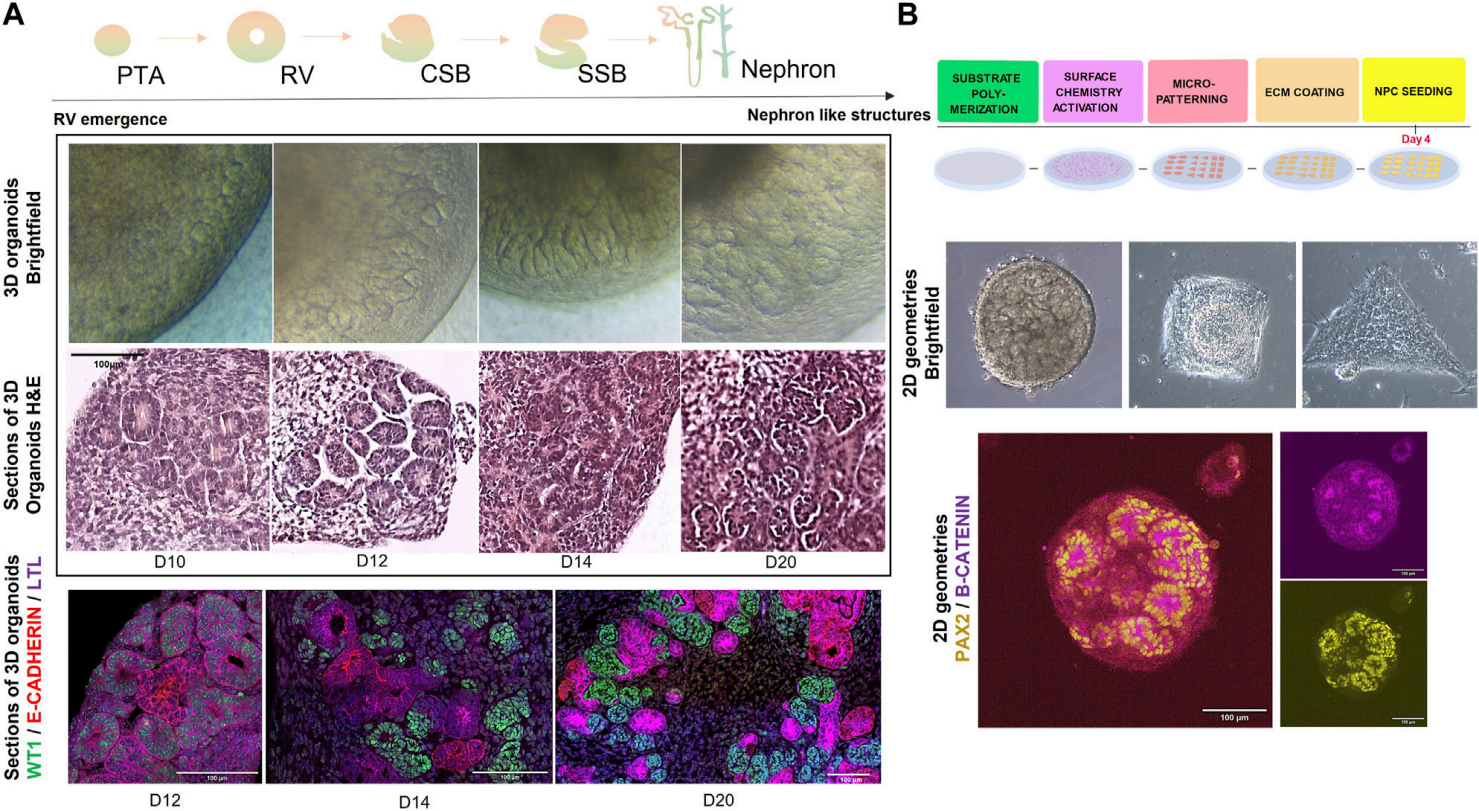
**(A)** (top) Schematic representation of steps in nephrogenesis: PTA (pre-tubular aggregates) -> RV (renal vesicles) -> CSB (comma-shaped body) -> SSB (s-shaped body) -> nephron. (middle) Brightfield and H&E representations of 3D kidney organoids during *in vitro* nephrogenesis (from day 10 until day 20 of differentiation). Scale bar = 100 µm. (bottom) Immunofluorescence microscopy images depicting stages of nephrogenesis in 3D kidney organoids>: WT1 (Wilms Tumour 1) in green, E-CADHERIN in red and LTL in magenta. Scale bar = 100 µm. **(B)** (top) Schematic representation of a stepwise methodology for generating micropatterns on substrates. Briefly, substrates of interest (PDMS, PAA) are polymerized to a rigidity mimicking embryonic stiffness (1–100 kPa), functionalized and micropatterned (for example, with photoactivation). Then the substrates are decorated with an ECM protein of interest (e.g., fibronectin, collagen, etc.,) upon which progenitors can be seeded for further 2D culture under geometric confinement. (middle) Brightfield microscopy images of hPSC-derived kidney organoids at day 9 of differentiation, subjected to geometric confinement (circles, squares and triangles of 0.07 mm^2^ area). (bottom) Immunofluorescence microscopy image of hPSC-derived kidney organoids at day 10 of differentiation, marked by PAX2 in yellow and B-CATENIN in magenta. Scale bar = 100 µm.

A major advantage of using *in vitro* kidney organoid systems to model organ development and CAKUT is that they can be interrogated *via* a variety of functional, physiological, and molecular techniques. One of the simplest and most accessible techniques is morphological analysis by brightfield microscopy, where structures like RVs, complex nephron like structures and cysts (in case of disease modelling) can be roughly identified during the differentiation protocol (Taguchi and Nishinakamura, 2017; Przepiorski et al., 2018; Garreta et al., 2019; Schutgens et al., 2019). Moreover, these structures can be subjected to more detailed histological or immunofluorescence analysis by confocal microscopy, where either whole organoids or tissue sections can be stained for markers such as *PAX2, WT1, LHX1, JAG1, E-CADHERIN, and NEPHRIN,* corresponding to different segments of the developing nephron (Figure 2A).

Single cell transcriptional profiling of human foetal tissue has been also a key tool in understanding the many intermediate transcriptional states of kidney development. Applying this technique to analyse kidney organoids at different stages of differentiation has also helped map the different cell types present more accurately, as well as provide a transcriptional comparison to human embryonic kidney development (Lindström et al., 2021; Wilson et al., 2022).

## Organ development and disease are guided by mechanical forces

Developmental biology has largely focused on discovering the function of morphogens and the molecular processes that drive embryogenesis and tissue formation. However, it has become more evident that tissue formation and morphogenesis do not solely rely on biochemical and genetic cues. In fact, these processes are also influenced by intrinsic and extrinsic biomechanical forces [reviewed in detail in (Heisenberg and Bellaïche, 2013; Goodwin and Nelson, 2021)]. On a subcellular level, cells can experience phenomena such as actomyosin contractility, membrane stretching and tension during cell division or upon exposure to very stiff microenvironments (Colombo et al., 2003; Krieg et al., 2008; Daley et al., 2011). Extracellularly, cells can undergo compression and extrusion during events like apoptosis and tissue remodelling (Yang et al., 2000; Eisenhoffer et al., 2012). On a much grander tissue scale, contractions, for example, by muscle fibres can be observed (Harrington, 1979). Extrinsic forces can even be observed on an organismal level, an example of which is the effect of gravity.

Nowadays, in developmental biology there has been a shift towards investigating how mechanical forces impact the development of tissues and understanding the physical mechanisms that facilitate morphogenesis. The idea that tissue morphogenesis is in fact a biomechanical process has been reviewed by pioneers in the 1980–1990 s (Wolpert, 1981; Beloussov et al., 1994). In fact, they brought forward the ideas that biochemical and mechanical morphogenesis within the developing tissue are not necessarily distinct, as the physical properties of local regions can affect the shape of morphogen fields and the movement of cells can alter how tissues respond to molecular gradients.

Quite like biochemical signalling molecules that instigate an intracellular sequence of events leading to changes in gene expression, mechanical stimuli can trigger a similar cascade. Here, cell adhesion complexes localized on cell membranes can adhere to components of the extracellular matrix (ECM) and create connections like cell-to-ECM and cell-to-cell junctions that are necessary for transmitting the biomechanical signals towards the nucleus and ultimately affect cell function. This ability of cells to sense their mechanical microenvironment is termed *mechanosensing*, while the signalling cascade of events is known as *mechnotransduction*. Cell fate decisions that are ultimately influenced by the cell’s mechanical microenvironment include migration, differentiation, proliferation, and cell death, among others.

During embryo development and organogenesis, the composition, stiffness and viscoelasticity of the ECM represent key factors previously reported to influence stem cell fate and differentiation [reviewed in detail in (Guilak et al., 2009; Elosegui-Artola, 2021)]. Indeed, *in utero*, the embryonic microenvironment contains a *plethora* of ECM proteins (such as fibronectin, collagen, laminin, etc.,) and a highly viscoelastic matrix (ranging from 0.1 to 100 kPa in stiffness) which has been shown to influence the fate of stem cells within the forming tissue (Guilak et al., 2009; Rozario and DeSimone, 2010; Chaudhuri et al., 2020).

A seminal example of this *phenomena* is the effect of ECM elasticity on lineage specification of mesenchymal stem cells (MSCs). Studies have shown how upon exposure to soft matrices, these multipotent MSCs can be driven to the adipogenic lineage, while stiffer microenvironments can induce osteogenesis in the same naïve stem cells (Engler et al., 2006; Guilak et al., 2009; Huebsch et al., 2010). Vascular progenitors *in vitro* have been shown to differentiate into endothelial lineage when exposed to soft microenvironments, while stiff matrices guide them to the smooth muscle cell fate (Wong et al., 2019). Specific ECM conditions are also crucial for the maintenance of tissue homeostasis. For example, the maintenance of mammary gland tissue homeostasis requires a soft and laminin-rich microenvironment (Alcaraz et al., 2008).

At the same time, stiffer matrices with altered ECM protein composition/density are typically conditions that support tumour growth and metastasis (Wullkopf et al., 2018; Bauer et al., 2020). A fascinating study on obesity and cancer invasion has shown how changes in the adipose tissue microenvironment, such as stiffening of the ECM from enrichment of myofibroblasts in obese mice, can increase the malignant potential of breast cancer cells (Seo et al., 2015; Ling et al., 2020). This study demonstrates the effect of ECM stiffness on tumorigenesis and the importance of mechanical stimuli on cell fate decisions (Elosegui-Artola, 2021). Meanwhile, the viscosity vs. elasticity of the ECM is becoming increasingly recognized as an additional modulator of cell fate decision (Chaudhuri et al., 2020; Elosegui-Artola et al., 2022). Matrix viscoelasticity has been demonstrated to guide single cell behaviour, as well as collective behaviours of spheroid/organoid growth and differentiation (Elosegui-Artola et al., 2022). Apart from the biophysical properties of the ECM, fluid flow in the extraembryonic environment has been shown to influence cell behaviour too [reviewed in (Freund et al., 2012)]. Briefly, fluid flow has been previously shown to influence left-right symmetry breaking during organ development (Tanaka et al., 2005), regulation of vascularization and sprouting (Song and Munn, 2011), and normal renal tubule function (Nauli et al., 2003).

The effect of fluid flow has also been explored on kidney organoid differentiation *in vitro* (Homan et al., 2019). By applying a fluid flow, Homan et al. were able to enhance kidney organoid vascularization and stimulate the maturation of podocytes, a specialized cell type in the kidney. The stiffness of the extracellular environment has also been explored in embryonic kidney differentiation. Garreta et al. and others have shown how a soft synthetic hydrogel can mimic the *in utero* mechanical microenvironment and enhance the maturation of kidney organoids cultured *in vitro* (Garreta et al., 2019; Ruiter et al., 2022). These studies highlight the importance of mechanical stimuli in regulating the differentiation of hPSCs into renal lineages and the generation of mature kidney organoids.

In adult kidneys, Choudhury et al., have explored the role of mechanical forces that drive fluid transport across the tubule epithelial cells. They described differences in fluid flux and pressure gradients in human healthy kidney cells vs. autosomal dominant PKD cells, which shed light on the importance of mechanical forces in kidney function and their implications in pathophysiological conditions (Choudhury et al., 2022). In efforts to understand PKD pathophysiology better, Cruz et al. have looked into the role of the microenvironment on disease progression. PKD is associated with the formation of fluid filled cysts from kidney tubular epithelia. The authors have identified that the microenvironment of tubule organoids can substantially increase or decrease the probability of cyst formation (Cruz et al., 2017).

These findings have significant implications for the development of new models for studying kidney development and disease, as well as the potential use of human kidney models in regenerative medicine.

All these studies demonstrate how mechanical input from the surrounding environment and the cell’s ability to mechano-sense these signals contribute to cell fate decisions during healthy tissue development and disease evolution. Nowadays, insights on how these mechanical signals are sensed and internalized by the cells to elicit a genetic response are being slowly uncovered (Discher et al., 2005; Heisenberg and Bellaïche, 2013; De Belly et al., 2022).

## State-of-the-art- techniques to interrogate organoid mechanobiology

### Mimicking the extraembryonic microenvironment

The discovery of Matrigel (Orkin et al., 1977), a natural ECM substitute composed of basement membrane proteins extracted from a murine tumour, has kickstarted an exponential growth of *in vitro* research (Kubota et al., 1988; Kibbey et al., 1992). This ECM surrogate has been extremely useful in assays of cell invasion and angiogenesis, formation of spheroids and the growth of organoids (Barker et al., 2010; Huch et al., 2013; Stange et al., 2013; Wang et al., 2017). However, in the age of translational research, where organoid systems could be potentially used for organ disease modelling and drug screening, the poorly defined and cancerous nature of Matrigel makes it unattractive for use. Furthermore, batch-to-batch differences in Matrigel production severely impact the extent of organoid differentiation and thus hamper comparative studies. For these reasons, the organoid field quickly started deriving well-defined and synthetic materials towards the generation of organoid models suitable for disease modelling and interrogations.

The progress in the field has led to the definition of substrates of tuneable stiffness and/or viscoelasticity as well as protein composition. Natural substrates include the derivation of biomaterials from decellularized ECM, the use of natural ECM proteins such as collagen or alginate. Interestingly, several works have demonstrated the use of synthetic hydrogels, such as polyacrylamide (PAA), polydimethylsiloxane (PDMS), and polyethylene glycol (PEG), among others. In this section the use of natural and synthetic substrates for organoid generation and disease modelling applications will be revisited.

### Natural substrates

Decellularized ECM (dECM) has the advantage of maintaining the natural architectural composition of tissues *in vivo*, making it a perfect candidate for long term maturation of hPSC-derived organoids *in vitro* [reviewed in detail in (Garreta et al., 2017)]. Depending on the de-cellularization treatment, dECM-derived scaffolds can maintain the mechanical and biological properties of the native tissue, allowing for cell attachment, migration and differentiation of new cells introduced into the empty scaffold. This approach has been successfully used for liver, intestinal, pancreatic and kidney-derived cells, among others (Orlando et al., 2013; Guyette et al., 2014; Batchelder et al., 2015; Hong et al., 2018). However, the process of using dECM scaffolds still faces several challenges: variations in decellularization protocols (dependent on tissue origin), difficulty of accurately re-populating the dECM with different cell types present in the native tissue, need for oxygen perfusion in areas of thicker tissue formation, lack of sufficient human material/dependence on organ donation, among others.

Naturally occurring ECM proteins, such as collagen or laminin can also be used to generate scaffolds for tissue engineering purposes [reviewed in (Glowacki and Mizuno, 2008)]. Collagen scaffolds are made up of collagen molecules (most commonly types I, II, and III) that, when covalently bound to one another, are termed collagen fibrils (Dong and Lv, 2016). These fibrils are further grouped into bundles, also known as fibres. The collagen fibres eventually provide the entire substrate with a specific biomechanical architecture that has been shown to support cancer cell migration, metastasis, and the maintenance of a few types of organoids (Wang, 2005; Han et al., 2016; Kopanska et al., 2016; Jee et al., 2019). However, collagen-based matrices are often not enough to fully support the development of tissues and require additional support from co-culture cells and additional ECM proteins (Wang et al., 2016).

Organoids have also been grown in natural polysaccharides like alginate, an inexpensive, biocompatible and readily-tuneable substrate suitable for biological application [reviewed in (Cattelan et al., 2020; Chen et al., 2020)]. Several studies have demonstrated the use of different modifications of alginate to study the growth and development of organoids of brain, kidney, lungs, intestine and pancreas (Wilkinson et al., 2018; Capeling et al., 2019; Liu et al., 2020; Geuens et al., 2021; Cassel De Camps et al., 2022). However, much like Matrigel, alginate is a biologically-derived material, meaning that it is also subjected to batch-to-batch variability (Fu et al., 2010).

### Synthetic substrates

Synthetic polymers, on the other hand, allow a precise control over the mechanical, chemical and structural elements of the substrate. By adjusting the composition of synthetic substrates, it becomes possible to mimic the viscoelasticity, porosity and density of certain tissues and study the effects of mechanical and chemical cues on cell fate decisions.

Polyethylene glycol (PEG) is a nontoxic and biocompatible polymer, increasingly utilized for cell culture and controlled release of biomolecules within *in vitro* tissues (Mellott et al., 2001; Drury and Mooney, 2003) [see in more detail in (Lin and Anseth, 2009)]. It is a highly tuneable substrate, adjustable via a variety of cross-linkers and functional groups, making it a unique ‘blank substrate’ for bioengineering purposes. PEG hydrogels allow the encapsulation of entire cell clusters/organoids, and have been shown to support a wide variety of cell types in culture (Lutolf et al., 2003; Moon et al., 2010; Phelps et al., 2012; Chaudhuri et al., 2016; Gjorevski et al., 2016; Ng et al., 2017).

Polyacrylamide (PAA) hydrogels can be tuned to a wide variety of stiffnesses (ranging from 0.2 kPa up to 200 kPa) depending on the compositional ratio of acrylamide and bis-acrylamide. In 1997, using this synthetic hydrogel, it was demonstrated for the first time that substrate stiffness can indeed affect cell adhesion, spreading and migration (Pelham and Wang, 1997). Following that, PAA and other synthetic materials became widely utilized to study the effects of stiffness as a mechanical cue on cell fate decisions (Engler et al., 2006; Wen et al., 2014).

Polydimethylsiloxane (PDMS), a material primarily intended for microfluidic applications (Duffy et al., 1998) has been gaining increasing recognition for cell culture purposes in mechanobiology and bioengineering (Brown et al., 2005; Chuah et al., 2015). Its tuneable stiffness, transparent nature, minimal autofluorescence and ease of use have made it a suitable candidate for studying cell-biomaterial interactions and understanding the effects of substrate stiffness on cell fate decisions (Mata et al., 2005). Here, techniques for surface chemistry activation have made it possible for cell attachment on the originally highly hydrophobic surface of the polymer.

Ultimately, the use of such versatile biocompatible natural and synthetic materials allows a much closer resemblance of *in vitro* culture conditions to natural *in vivo* tissue microenvironments. Moreover, since synthetic tuneable materials allow a tight control of substrate stiffness, porosity, density, etc., it becomes possible to perturb the mechanobiological environment of cells and question the way tissues respond to mechanical input from their environment.

### Measuring forces exerted by tissues and cells

Thus far, many studies have evidently demonstrated how mechanical stimuli like stiffness, viscoelasticity and fluid flow can influence cell fate decisions (Vining and Mooney, 2017), and have therefore prompted mechanobiologists to investigate methods for measuring the physical forces felt and exerted by cells. To this day, there are a variety of techniques that allow the measurement of forces exerted directly by cells [reviewed in (Roca-Cusachs et al., 2017)], techniques to probe cellular responses to forces exerted exogenously on them and techniques to measure the stiffness and rheology of certain tissues [reviewed in (Campàs, 2016; Polacheck and Chen, 2016)] (see Table 1).

**TABLE 1:** List of techniques to measure cellular and tissue level forces.

Technique	Principle of action	Citations
Traction Force Microscopy (2D & 3D TFM) 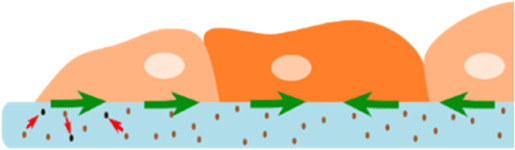	• Uses compliant viscoelastic substrate and fluorescent microbeads • Cells seeded on top (2D) or embedded within (3D) a hydrogel • Cells exert forces that generate a deformation in the hydrogel. Gel deformation allows the generation of a displacement map • Computational methods can be used to obtain traction forces exerted by the cells	Dembo and Wang (1999), Butler *et al.* (2002), Trepat *et al.* (2009), Han *et al.* (2015), Bergert *et al.* (2016)
Cantilevers (AFM) 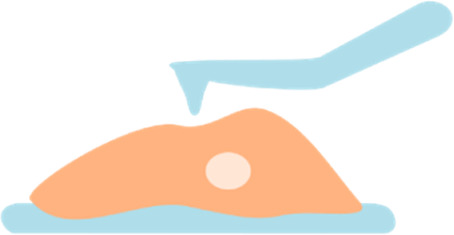	• Allows the application and quantitative measurement of mechanical force on a cell/tissue • Based on the use of a calibrated cantilever with known mechanical properties that can deflect when in contact with a surface (for example, cells) • The displacement/bending of the cantilever can be used to calculate the forces exerted by the cell as well as determine the stiffness/rheology of the tissue	Krieg *et al.* (2008), Badique *et al.* (2013), Taubenberger et al. (2014), Schillers *et al.* (2017)
Micropillars 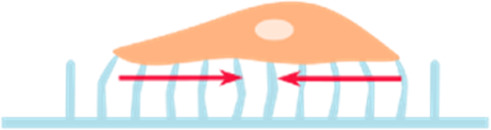	• An array of cylindrical columns made of a deformable elastic substrate with cells on top • Depending on the stiffness of the pillars, cells can exert forces that are able to deform these pillars • Mapping the deformations of the pillars and comparing to their undeformed state, allows to calculate the forces exerted by the cells	Tan *et al.* (2002), Rajagopalan and SaifTaher (2011)
Droplets & Inserts 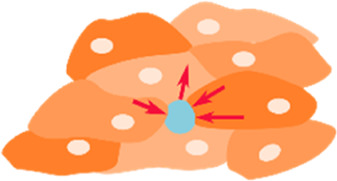	• Microdroplets of deformable compliant viscoelastic materials • Can be used to evaluate the mechanical properties of a tissue *in vivo* • Can be injected into a tissue of interest, imaged, and compared to their undeformed state, to gain a map of local tissue stresses	Träber *et al.* (2019), Mongera *et al.* (2023)
Fluorescence Resonance Energy Transfer (FRET) sensors 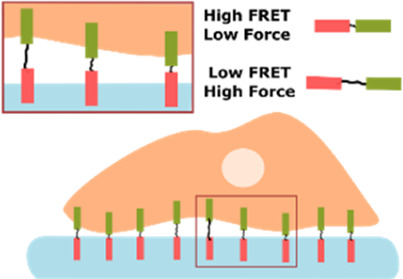	• Used to study cellular forces like tension between cell, and their surround ECM. • Contain two fluorescent proteins (energy acceptor and donor), linked by a tension sensitive linker (spring) • When a force is applied to the linker, the distance between the fluorophores changes, resulting in a change in energy transfer. This change can be recorded by fluorescence lifetime imaging microscopy (FLIM) and translated to tension measurements	Grashoff *et al.* (2010)
Micropipette aspiration 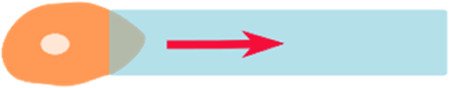	• Involves a glass micropipette of 0.5–10um diameter to apply a controlled suction force to a single cell • The suction force generates an indentation in the cellular membrane • The deformation of the membrane can be measured through microscopy and can help determine other mechanical properties of the cell: elasticity, viscosity, and tension along the membrane	von Dassow et al. (2010), Maître *et al.* (2015), Irianto *et al.* (2016)
Laser ablation (LA) 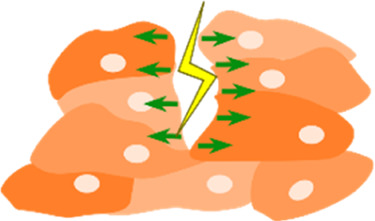	• Uses a focused laser beam to dissect/disrupt a local tissue or cellular structures • Can be specifically targeted at mechanical components of the cell: focal adhesions, cytoskeletal components, intercellular junctions, etc. • The movement/deformation of tissues can be used to infer whether the tissue before was in a tense or compressed state, and to further calculate the extent of these forces	Kiehart *et al.* (2000), Samarage *et al.* (2015)
Optical Tweezers (OT) 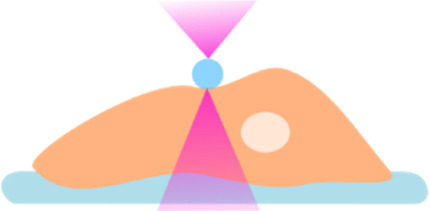	• Uses focused laser beam to trap a microbead that can be used to apply force to another subject, such as a cell • Position of the laser beam can be manipulated to control the forces being exerted by the trapped object • Resulting deformations on the cell membrane can be measured: cell indentation, cell stretching, and active rheology	Zhang and Liu (2008)

Mechanical forces are involved in various biological processes, including cell division, tissue growth, and organ function. The ability to measure these forces *in vivo*, *in vitro*, and *ex vivo* can provide valuable insights into the underlying mechanisms of these processes.

Most techniques in mechanobiology rely on the deformation of a viscoelastic material of known mechanical properties: traction force microscopy (TFM), micropillars, microdroplets/micro-inserts, cantilevers/atomic force microscopy (AFM). These techniques can be reliably used to measure cellular forces *in vitro (most)* (Dembo and Wang, 1999; Zimmermann et al., 2000; Tan et al., 2002; Rajagopalan and SaifTaher, 2011; Harris et al., 2012)*, ex vivo* (AFM) (Moore et al., 1995), and even *in vivo* (AFM, microdroplets) (Feroze et al., 2015; Mongera et al., 2023).

Other techniques, like FRET tension based sensors, rely on molecular linkers/fluorophores that can allow the measurement of tensile forces between cells and their extracellular environment (Grashoff et al., 2010) and even at the level of the nucleus (Andreu et al., 2022).

The mechanical properties of whole embryos (*xenopus*, zebrafish) at early stages of development have been widely explored with the use of micropipette aspiration and laser ablation techniques (von Dassow et al., 2010; Chaigne et al., 2013; Maître et al., 2015). Here the viscoelasticity of the tissue, the tension and contractile forces maintaining the whole structure together have been investigated (Rauzi et al., 2008; Samarage et al., 2015). While the mechanical properties of early embryos have been extensively studied in animals, the challenges and limitations associated with studying human embryos have made it more difficult to investigate these properties in humans. Due to the scarcity of human embryonic material and the ethical regulations forbidding the culture of human embryos past day 14 of development, the *in vivo* study of mechanical forces within developing organs seems practically impossible.

While these techniques can effectively allow the mechanical probing of living tissues, it is unfortunately not possible to assess *in vivo* the cellular stresses/forces acting within and upon the developing organs in humans.

## Combining experimental mechanobiology techniques with organoid technology to advance fundamental knowledge on organ development and disease

To this day, mechanical stimuli like substrate stiffness, physical confinement and fluid flow have been clearly proven to affect cell fate decisions in 2D-*in vitro* environment (s) [see reviewed in (Mammoto et al., 2012; Wagh et al., 2021)]. *In vivo*, however, tissues are far more complex, where viscoelasticity plays a key role dictating cell behaviour (Cheng et al., 2008; Geerligs et al., 2008; Safshekan et al., 2017). Here, a multitude of cellular and extracellular components, including the cytoskeleton, ECM proteins, membrane adhesion complexes, cellular cortex, among others, can influence the biomechanical signalling pathway and lead to variable cellular responses in time and space. Moreover, the practical difficulty of accessing organs and tissues during development *in vivo* increases the complexity of studying their mechanics. All this makes the measurement and probing of the mechanical environment of living tissues *in vivo* far more complicated.

However, with the rise of organoid technologies it has become possible to mimic embryonic organ development *in vitro* more closely and to investigate the mechanical changes occurring during early steps of organogenesis in a dish. By combining organoid technology with experimental mechanobiology techniques, it becomes possible to study the mechanical properties of cells and tissues and how they influence organ development and disease in a more controlled and physiologically relevant environment. This can provide new insights into the role of mechanical forces in these processes and help develop new therapeutic strategies for a variety of diseases.

There are several ways to study the mechanobiology of organoid development: through bioengineering of their microenvironment (exposure to synthetic and natural hydrogels, micropatterning, microfluidics devices, etc.), application of mechanobiology techniques (micro-rheology, TFM, optical tweezers, laser ablation, etc.,) and genetic manipulation of key genes involved in mechanosensing and transduction pathways (YAP/TAZ—HIPPO pathway, WNT signalling pathway, SRF pathway).

### Mimicking the extraembryonic environment through synthetic hydrogels

To closely mimic and bioengineer the extraembryonic environment, organoids can be encapsulated in hydrogels of varying stiffness, composition, and viscoelasticity. The effect of these substrates can be evaluated qualitatively by the rate of maturation (expression of markers by immunofluorescence microscopy, histology, gene expression) and attainment of certain organ-specific functionality.

Interestingly, studies have shown that separate stages of organoid development can require varying mechanical inputs from their surrounding ECM. For example, a fibronectin-rich and stiff ECM is a requirement for intestinal stem cell expansion, while for their differentiation and formation of intestinal organoids, a softer laminin-decorated matrix is preferred (Gjorevski et al., 2016; Broguiere et al., 2018). Similarly, encapsulation of kidney organoids into synthetic PEG-based hydrogels during later stages of differentiation has been shown to reduce undesirable fibrosis and promote a slight further maturation in organoids generated from hPSCs (Geuens et al., 2021; Ruiter et al., 2022). Similar studies have also been demonstrated in organoids of other organs, for example, the brain (Cassel De Camps et al., 2022).

While encapsulation in elastic hydrogels can provides the organoid with a mechanically stimulating surrounding, synthetic hydrogels can also physically limit the expansion and morphogenesis of the organoid during development (Wang and Heilshorn, 2015; Ranga et al., 2016). To overcome this limitation, PEG-based hydrogels can be effectively engineered to dynamically rearrange the constituting bonds, so that the surrounding synthetic ECM can adapt to the expanding organoid and support its morphogenesis (Chrisnandy et al., 2022). Such synthetic matrices allow a more reliable system to follow the mechanics of organ formation over time.

To quantitatively study the mechanical forces being exerted by organoids during development, fluorescent microbeads incorporated into a hydrogel of known viscoelastic properties can be used for traction measurements (TFM) (Bergert et al., 2016). 3D TFM on encapsulated organoids can provide valuable information on the directionality of tissue growth, the stresses and tensions necessary for the maintenance of their shape and the internal pressures that guide growth in specific directions (Steinwachs et al., 2016; Ban et al., 2018; Broguiere et al., 2018; Mulligan et al., 2019). Moreover, certain studies on morphogenesis of epithelial tissues have demonstrated how three-dimensionality is not always necessary for guiding tissue growth and differentiation (Pérez-González et al., 2021). In 2D, organoid systems can gain a degree of simplicity that allows a more reliable measurement of cellular forces (2.5D TFM), without compromising the morphogenetic process [reviewed in (Matejčić and Trepat, 2023)].

Alternatively, cellular forces exerted from within the developing organoid can also be studied by measuring deformations on hydrogel-based microdroplets inserted into organoids. The deformations caused on the microdroplets can be studied to identify the local forces exerted by cell structures (Girardo et al., 2018). Although this technique has been predominantly used to study forces within developing embryos (Träber et al., 2019; Mongera et al., 2023), under the same conditions, they could be used within organoids to study local forces arising during organogenesis. Indeed, further cell-specific functionalization with antibodies of the microbeads could allow for the control of localization of the microbeads within the organoids, allowing for force measurements at specific structures.

While such methods offer a great general overview of the types of forces present in and around tissues, it is crucial to stay aware of their limitations. Traction force measurements in both 2D and 3D settings often assume the surrounding matrix to be linearly elastic, neglecting inelastic effects. Relying solely on elastic assumptions may indeed lead to inaccuracies in calculating forces, especially when dealing with large 3D tissue structures such as organoids and taking into consideration timeframes involving significant remodelling of the ECM around the cells.

While the pursuit of more accurate force measurement techniques should be prioritized, currently available techniques based on displacement measurements can still provide a thorough overview into the mechanics of tissue systems (Clark et al., 2022). In the case of inelastic materials, where relaxation/trypsinization based force calculations are unfeasible, tissue and cell dynamics can still be reliably monitored (Godeau et al., 2022; Yamaguchi et al., 2022). Nevertheless, in this young field, there are still several unexplored avenues for mechanobiological research in organoid systems, one of which could involve, for example, load history quantifications.

### Using geometry and microfluidics to guide organoid morphogenesis

While substrate stiffness does indeed play a crucial role in *in vitro* tissue morphogenesis, recent studies have additionally been pointing towards the effect of geometry on tissue organization and differentiation. Seminal studies from Warmflash et al. have demonstrated how a simple geometrical confinement is enough to trigger self-organization in hPSCs (Warmflash et al., 2014). Others have explored how these geometries generate nodes of cell adhesion tension, that have been shown to guide mesoderm specification in hESCs (Muncie et al., 2020). Geometry has also been demonstrated to influence the morphogenesis past gastrulation in organoids *in vitro*. In kidney morphogenesis, geometries can be used in combination with ECM mimicking substrates to explore the effect of mechanical input on *in vitro* differentiation (Figure 2B). Curved geometries have improved brain region specification of human cerebral organoids, while controlled circular patterns seem to promote cardiac beating in human cardiac organoids (Ma et al., 2015; Sen et al., 2021). Micropatterning geometries in 2D also allow a higher control over tissue morphogenesis in events, like the formation of the neural tube (Karzbrun et al., 2021). Karzbrun et al. have beautifully demonstrated how a precise control of pattern size, shape and 2D hPSC monolayer density can lead to the process of neural tube folding and lumenogenesis. In a similar manner, Gjorevski et al. have engineered geometrically defined microwells that allow a controlled generation of intestinal crypts and villi from stem cells (Gjorevski et al., 2022). Such approaches are paving the way to effectively studying human synthetic morphogenesis *in vitro.*

During organogenesis, certain organs such as the brain and the gut, undergo a high level of tissue folding. These phenomena are particularly fascinating: during the start of morphogenesis, these organs start their development from 2D monolayers of epithelia, which over time are subjected to buckling and folding, giving rise to complex structures such as the gyri of the brain and villi of the intestine [reviewed in detail in (Nelson, 2016)]. With the rise of organoid technologies, the mechanical and physical stimuli guiding such events, for example, the folding of the brain, have been effectively explored (Karzbrun et al., 2018). Here, the physical forces accompanying morphogenesis can be studied by engineering a microchip/microfluidics model of the organ of interest and measuring angles of bending/bulging/deformations/flow rates during development [reviewed in (Polacheck et al., 2013)].

In the framework of kidney development, the mechanisms involved in the branching of the UB and the organization of collecting tubules has been a topic of increasing interest. While it has been well established that the branching of the UB into the MM is driven by morphogenetic signalling in the developing kidney, the nature of how biomechanical defects lead to CAKUT phenotypes is still an open question. In this regard, other studies have investigated the tissue forces and dynamics of tubule organization under geometric confinement of the bud tips at the surface of the developing kidney (Viola et al., 2022; Prahl et al., 2023). Indeed, these works provided a model that explains the packing of the collecting duct tubules in murine kidney development during healthy and UB defect scenarios. While there is no doubt on the effect of morphogenetic signalling on healthy kidney development, studies such as Prahl et al. shed light on the importance of tissue forces and dynamics in the overall organization of such complex organs (Prahl et al., 2023).

### Using mechanosensing and mechanotransduction machinery to investigate organoid morphogenesis

While the review has so far focused on the study of mechanics of organoids from a top-down approach (i.e., manipulating the extracellular environment to elicit a cellular response from transcriptional activation), such mechanics can also be studied bottom-up. By modulating key players of the mechanotransduction signalling pathways, it is possible to interrogate how mechanosensitive protein activation and gene transcription influences tissue morphogenesis [mechanosensitive mechanisms in transcription have been carefully reviewed in (Mammoto et al., 2012)].

When cells are exposed to an external mechanical stimuli, the first line players that initiate the mechanotransduction pathway are mechanosensitive membrane proteins (Ranade et al., 2015). These include the mechanosensitive ion channels of the Piezo protein family, an example of which is Piezo1. In the past decade, several studies have investigated the role of Piezo1 as a mechanotransducer and have identified its role in vascular, neural and bone development (Pathak et al., 2014; Ranade et al., 2014; Li et al., 2019). In the urinary system, Piezo proteins are highly important in sensing shear stress and wall tension to ensure proper flitration (Li et al., 2022). While the exact implication of these proteins in kidney development and disease are yet to be explored, the potential importance of these mechanosensitive ion channels in the urinary tract cannot be ignored (Dalghi et al., 2019).

The high sensitivity of these membrane proteins to instigate mechanotransduction further down the cell, makes it a potent candidate for tissue engineering purposes. By regulating such ion channels through, for example, genetically induced light activation, it could become possible to control cellular responses in the absence of mechanical stimuli.

Further down the mechanotransduction pathway are mechanosensitive transcription factors that inititate the activation of genes in the nucleus to prompt a cellular response to mechanical stimuli.

Yes associated protein (YAP)/Transcriptional coactivator with PDZ-binding motif (TAZ) are one of the most known mechanosensitive transcriptional regulators, which evidently play an important role in organogenesis (Mosqueira et al., 2014; Piccolo et al., 2014). They are modulated by both biochemical [HIPPO signalling (Meng et al., 2016)] and mechanical [ECM stiffness, shear stress, stretching (Aragona et al., 2013; Elosegui-Artola et al., 2016) cues, depending on which they are localized either in the cytoplasm or nucleus.

In the kidneys, TAZ activity has been shown to be associated with cystogenesis in PKD, implying the important effect of mechanotransduction during kidney disease progression (Lee et al., 2020).

At the level of stem cells, depletion of this key transcription factor (YAP) has been shown to result in a loss of pluripotency, while constituent expression leads to maintenance of stemness (Lian et al., 2010). Studies have shown that control of stem cell fate through mechanical stimuli such as substrate stiffness is dependent on YAP localization (Mosqueira et al., 2014; Musah et al., 2014; Totaro et al., 2017), where stiff matrices promote nuclear localization by stretch of the nucleopores (Elosegui-Artola et al., 2017; Andreu et al., 2021; 2022). Such seminal studies imply that by directly regulating YAP/TAZ localization, it is possible to effectively direct mechnotransduction, and in the end cell fate, without the application of external environmental forces.

These studies demonstrate the importance of understanding the mechanosensing and mechanotransduction machinery in cells as a bottom-up approach to control morphogenetic events during development. Here the use of human PSC-derived organoids can serve as a framework to question the mechanical aspects of organ development, where bioengineering advances can aid in the realization of these studies in a controlled and reproducible manner.

## Conclusion

Altogether, accumulated findings within the last decades have revealed the immense value of hPSC-derived models as a platform on which organogenesis could be recapitulated. By investigating the specification of stem cells towards the kidney lineage *in vivo*, researchers gained an in-depth knowledge regarding the principles governing nephrogenesis, an invaluable asset to ultimately recreate this process *in vitro*. While *in vitro* models of human kidney organogenesis can to some extent faithfully recapitulate the organ’s microanatomy and physiology, there are still major limitations in reproducibility, control of cell type, composition, and lack of vascularized and innervated structures. Here, by acknowledging the mechanical microenvironment of *in vivo* tissues during development, and by closely mimicking these external cues, it becomes possible to drive organoid maturation a step further and gain a better understanding of organogenesis and disease progression.

While current advances in bioengineering and mechanobiology are paving new strategies for studying and guiding tissue morphogenesis *in vitro*, there is still a long way to go. In order to guide cells in the direction of organ development, biochemical and mechanical signalling cascades within and between cells need to be perfectly coordinated to navigate through the complex dynamics of tissue patterning. Hence in kidney organogenesis, more consistent efforts are needed to truly understand the underlying biomechanical machinery and how it works in concert with the genetic program. Only then will it be possible to engineer functional kidney tissues.
